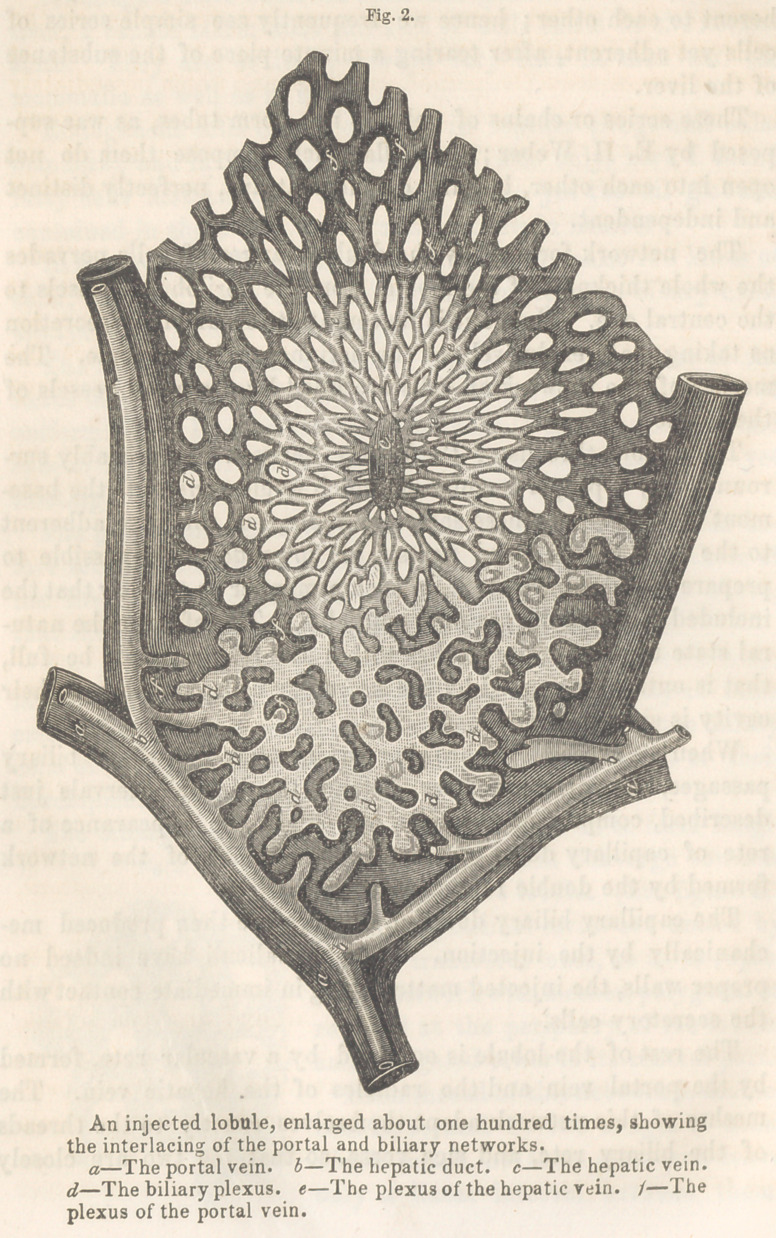# M. A. Lereboullet upon the Intimate Structure of the Liver and the Nature of the Alteration Known under the Name of Fatty Liver

**Published:** 1854-04

**Authors:** 


### *M. A. Lereboullet upon the Intimate Structure of the Liver and the Nature of the Alteration known under the name of Fatty Liver. Portal Prize Essay.*

(Translated for the Medical Examiner by JAS. E. RHOADS, M. D., Resident Physician at the Pennsylvania Hospital.)

M. Lereboullet, in a monograph upon the above subjects, after an extended and very lucid explanation of his views of the structure of the liver, as derived from its examination in man, and the different orders of animals, both in the adult and fœtal condition, and from micrometric measurements of the constituents of the lobules in injected preparations from the human subject, sums up the results of his investigations in the following general conclusions.

The organs which prepare the biliary fluid in all animals are cells; that is, hollow organic elements analogous to the utricles of vegetables.

The liver of the molluscs and of the crustaceæ, as the craw-fish, contain two kinds of cells, biliary and fatty.

These two kinds of cells multiply by endogenous generation. The fatty cells appear to be only transitory, and are transformed into biliary cells by the deposition of biliary granules within them, and the disappearance of the oily particles which they contained.

The liver of the vertebrate animals is composed of lobules, that is, of small aggregations of the secretory elements, which are so grouped as to form masses of variable dimensions, seldom exceeding two millimetres, or rather less than a line in diameter. These lobules are frequently more or less blended with each other. They are most readily distinguished in the liver of the hog, in which they are surrounded by a special capsule, in direct continuation with the capsule of Glisson. In the human liver the lobules are always more or less confounded with each other.

The two colors of the liver, which render visible its division into lobules, are not due to the existence of two distinct substances, nor to the greater or less accumulation of bile in the ducts, but depend solely upon the relative degree of repletion of the perilobular portal vessels, and of the hepatic veins which occupy the centre of the lobules.

When the stasis of the blood occurs in the portal veins, the periphery of the lobule is darker than the centre; the contrary state obtains when the portal veins are empty, while the central ones are filled with blood, as is seen in many pathological states, particularly in the fatty liver.

In the liver of the hog there exists, around each lobule, a true cellular envelope, which is easily demonstrated, and which separates the lobules perfectly from one another. The fibrous elements of this envelope are continuous with the cellular tissue surrounding the vessels, (capsule of Glisson). In man no trace of this lobular envelope can be demonstrated.

The hepatic lobule is, in itself, a small liver, composed of secretory cells, a capillary rete of afferent and another of efferent blood vessels.

The secretory or biliary cells of the vertebrate animals are, like those of the invertebrata, true utricles.

The opposite walls of these closed sacs are more or less pressed together, but may be distended and rendered ovoid by treating them with chloroform. The examination of fatty cells also shows that the oil is developed in their interior and distends their walls.

These cells habitually contain, 1st. A spherical nucleus, with a variable number of transparent, punctiform nucleoli. 2d. Grey or fawn colored granules, dispersed through the cell or accumulated in minute masses (biliary granules). 3d. Very small oil globules, scattered among the preceding.

The existence of these several elements in the interior of the cells is not constant. The nucleus is occasionally wanting, the biliary granules are not always aggregated, and the fatty vesicles are not always distinct.

The dimensions of the nucleus are nearly constant, yet I have frequently found nuclei much larger than ordinary, and which may be regarded as included cells.

Cells containing two nuclei of equal size are sometimes met with; this circumstance, rare in healthy livers, appears more commonly in certain cases of disease of this viscus.

Although I have observed some endogenous cells in the human liver, I cannot affirm that this sort of cells exists in the normal state. They are, at least, always very rare in man and the mammalia as well as in birds.

Endogenous cells exist positively in reptiles (frogs and salamanders), and in fish. In the liver of fish only, have I found fatty cells distinct from the biliary cells; yet the oil globules contained in these cells were small and few in number.

In the liver of the fœtus of mammals there exist two kinds of cells; fatty cells in great numbers, and endogenous biliary cells, always much smaller than the preceding.

The fatty cells which composed almost the entire liver of the fœtus of a rabbit of fifteen days, were filled with oil globules of uniform size. In a human fœtus at term, I could no longer find special fatty cells, but found some endogenous biliary cells yet existing.

The predominance of fatty cells in the liver of the fœtus before term, and their existence in the livers of fish and in those of the invertebrata, confirm me in the opinion announced above, that the fatty cells are the first state of the biliary cells.

The great number of endogenous cells, whether fatty or biliary, in the inferior animals and in the fœtus, and the rarity of these cells in the superior vertebrata, authorise us to regard the biliary cells of these latter as having attained the full extent of their development.

The biliary cells are joined by their ends so as to form longitudinal series which converge towards the centre of the lobule. (See figure 1.) These longitudinal series are united by shorter transverse ones, so as to represent a network with meshes, polygonal or rounded at the periphery of the lobule, and elongated towards its central part.

Fig. 1.

Each thread of this network is double, that is, formed by two ranges of cells, which touch at their sides and leave only a linear interval between them.

But these two ranges of cells are only in juxtaposition, separating easily by the slighest traction.

The cells which constitute the series are, on the contrary, adherent to each other; hence we frequently see simple series of cells yet adherent, after tearing a minute piece of the substance of the liver.

These series or chains of cells do not form tubes, as was supposed by E. H. Weber; the cells which compose them do not open into each other, but are, on the contrary, perfectly distinct and independent.

The network formed by the double ranges of cells pervades the whole thickness of the lobule, from the perilobular vessels to the central one. Hence it is inaccurate to speak of the secretion as taking place exclusively at the periphery of the lobule. The meshes of the network of cells are filled by the blood vessels of the lobule.

The double threads of the biliary network are probably surrounded by a proper membrane, which would constitute the basement membrane of these secretory tubes, but this is so adherent to the walls of the blood vessels, as to render it impossible to prepare and demonstrate it, in such a manner as to show that the included biliary cells are only epithelial. Therefore in the natural state these secretory tubes within the lobules would be full, that is entirely occupied by the secretory cells, and hence their cavity is simply linear.

When we succeed in throwing an injection into these biliary passages, the injected matter distends the linear intervals just described, compresses the cells, and gives the appearance of a rete of capillary ducts which takes the place of the network formed by the double ranges of cells.

The capillary biliary ducts of authors are then produced mechanically by the injection. These canaliculi have indeed no proper walls, the injected matter being in immediate contact with the secretory cells.

The rest of the lobule is occupied by a vascular rete, formed by the portal vein and the radicles of the hepatic vein. The meshes of this network adapt themselves exactly to the threads of the biliary rete, and vice versa, so that the two are closely interlaced. (See figure 2.) The mean diameter of the threads forming the meshes, and of the meshes themselves in either network, is .015 of a millimetre.

Fig. 2.

An injected lobule, enlarged about one hundred times, showing the interlacing of the portal and biliary networks.

*a*—The portal vein. *b*—The hepatic duct. *c*—The hepatic vein. *d*—The biliary plexus. *e*—The plexus of the hepatic vein. *f*—The plexus of the portal vein.

The threads of the blood-vessel network of the lobule are tubes with definite walls and not simply channels. The existence of the walls of these vessels can be demonstrated and their structure studied. The portal network occupies the periphery of the lobule; it is formed by small vessels which are given off, at short intervals, from the perilobular veins, and which immediately become capillaries. The meshes of this network are polygonal.

The network of the hepatic vein fills the central half of the lobule; its meshes are elongated, (see figure 2), and terminate in the central vein, or intralobular vein of Kiernan.

The biliary secretion does not then take place in a circumscribed portion of the lobule, as some authors have stated; at the periphery, according to some, and at the central portion according to others, but in every part of its thickness, since the secretory cells exist in all parts of it and are supplied at every point by blood vessels.

Every lobule has its axis traversed by a vein, the central vein, which either terminates in a cœcal extremity, or divides into several diverging branches. These central veins either unite to form a branch of the hepatic vein, or open directly into one of its branches upon which the lobule is placed.

Upon slitting open an hepatic vein, the orifices of these central veins may be seen, by the naked eye or by the aid of a lens, generally placed at the centre of the lobule, whose outline may be distinguished through the walls of the vessel.

The biliary canals which leave the lobules are always multiple. They arise from all points of the surface of the lobule, and after having united with each other many times, like the roots of a tree, leave the lobule and form one or more ducts, which, with the corresponding trunks of the portal vein and hepatic artery, are surrounded by a fibrous sheath, the capsule of Glisson.

The portal vein, after subdividing in the capsule of Glisson, furnishes ramuscles which encircle the lobules, but never form a perfect and complete vascular sheath around any one lobule, since each lobule receives numerous branches from the neighboring portal veins; and it is from the reunion of all these perilobular branches, that the more or less complete vascular circle is formed around each lobule, and from which the lobular portal rete takes its origin.

The hepatic artery, which everywhere accompanies the portal vein, does not concur in the formation of the lobule. Its ramifications are expended upon the walls of the vessels and in the capsule of Glisson.

They have a particularly abundant capillary distribution in the subperitoneal cellular tissue at the surface of the liver. This network, which the hepatic artery forms at the surface of the liver, does not differ from the rete of the subjacent portal vein. They have the same dimensions, are continuous with each other, and thus really constitute but one.

The blood of the hepatic artery does not appear to concur in the formation of the bile, or, at least, the part which it plays in this secretion is very secondary.

The walls of the hepatic duct, ductus choledochus, cystic duct and gall bladder, are lined by closed ovoid follicles, which form by their union small granular sacs, lying upon the exterior of the ducts, and furnished with an excretory canal which opens into the latter.

The closed follicles in these granular sacs are lined on their interior, by an epithelial formed of small granular spherical cells.

## Figures and Tables

**Fig. 1. f1:**
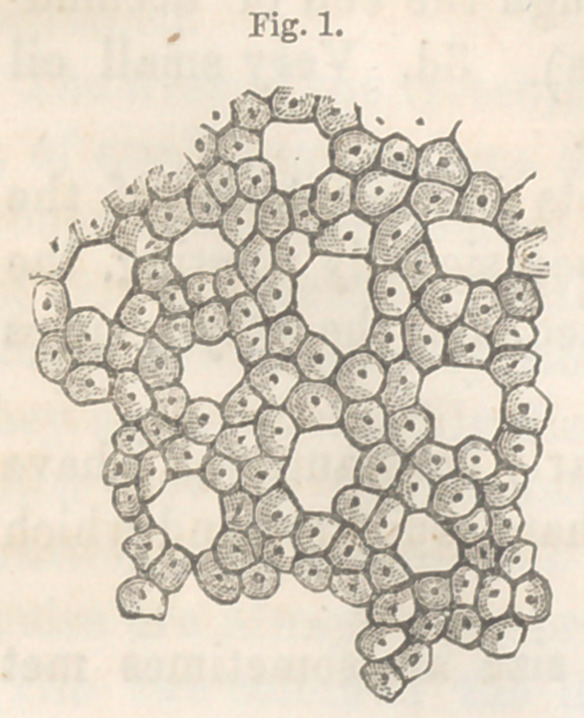


**Fig. 2. f2:**